# A Passive Magnetoelastic Radiation Sensor

**DOI:** 10.3390/s19224959

**Published:** 2019-11-14

**Authors:** Vincent Lamberti, David Mee, Peter Angelo, Jeffrey Preston

**Affiliations:** Consolidated Nuclear Security, LLC, Y-12 National Security Complex, P.O. Box 2009, Oak Ridge, TN 37831, USA; david.mee@cns.doe.gov (D.M.); peter.angelo@cns.doe.gov (P.A.); jeffrey.preston@cns.doe.gov (J.P.)

**Keywords:** passive radiation sensor, magnetoelastic sensor, wireless, sensor array

## Abstract

A passive gamma detection technology, consisting of a fielded sensor and a separate analysis system, is described. The sensor is a small cylinder, about 2.5 mm in diameter and 15 mm in length. It requires no onboard power sources or physical connections for power or data transfer, and retains its cumulative response to radiation. The sensor consists of an amorphous magnetoelastic wire held in a longitudinally-stressed state by a radiation-sensitive material. When the radiation-sensitive material is subjected to gamma radiation, it mechanically degrades, relaxing the stress on the wire and lowering the wire’s magnetic permeability. The changes in permeability are observed by switching the magnetic domains in the wire and measuring the reduction in the Faraday voltage as the stress is diminished. The analysis package is built around an excitation-detection coil set and can communicate wirelessly with the sensor through a metallic or nonmetallic barrier at distances up to about 25 mm. The sensor response is linear up to a dose of at least 7 kGy.

## 1. Introduction

Passive radiation sensors, which must be processed in some manner after exposure to be interpreted, have been employed since the earliest investigations into radioactivity. In 1895, Wilhelm Röntgen discovered that a cardboard screen painted with barium platinocyanide displayed a green fluorescence when placed in the path of X-rays generated in a Hittorf–Crookes tube [1,2]. A short time later, Röntgen built the first passive radiation sensor by replacing the barium platinocyanide screen with a photographic plate requiring development, and then created the first medical X-ray image by capturing the bones in his wife’s hand. Improvements rapidly followed, including sensitized plates, flexible films, and intensifying screens. Present-day passive radiation sensors take many forms, some bearing very little relation to Röntgen’s painted screen. Modern photographic emulsions are available as radiographic films for general imaging and as thicker nuclear emulsions for recording individual particle tracks [3,4]. Thermoluminescent dosimeters (TLDs) are based on materials that capture energy from ionizing radiation in the form of deeply-trapped electrons and holes, and release it as light when heated; the number of trapped charge carriers, and thus the amount of emitted light, is proportional to the absorbed dose [5]. Superheated drop or “bubble” detectors employ liquids that vaporize into tiny bubbles along particle tracks [6,7]. Neutron activation foils exhibit induced radioactivity after being subjected to a neutron field, and decay in a manner that can be used to determine the energy distribution of the incident radiation [8]. Recently, Létant and Wang [9] proved that semiconductor quantum dots can convert alpha radiation into visible photons.

Many sensor technologies have been based on magnetoelastic materials. Grimes [10,11,12] and his associates developed numerous variations of a sensor in which the principle of detection relies on changes in the resonant frequency of an amorphous ferromagnetic foil coated with a material responsive to specific chemical or biological analytes. Baimpos et al. [13] demonstrated that a sensor of this type, built with a Metglas 2826 MB ribbon coated with Bayhydrol-110 (a polyurethane dispersion in water/*n*-methyl-2-pyrrolidone), exhibits enhanced selectivity toward *o*-xylene and *p*-xylene against six other volatile organic compounds (*c-* and *n*-hexane, benzene, ethyl acetate, methyl ethyl ketone, and dichloromethane). Closer to the theme of this paper, Du et al. [14] developed a wireless resonance-based sensor that responds to radiation-induced changes in the viscosity of an acrylamide solution. Stoyanov et al. [15] described a remotely interrogable sensor consisting of an array of soft ferromagnetic thin-film structures attached to a polymer that swells or shrinks in the presence of a target; as the polymer deforms, it expands or contracts the distances between the magnetic films, modifying their magnetostatic coupling and switching behavior. Other published devices are directed toward measurements of fluid flow [16], pressure [17], and strain [18]. Very recently, Zhang et al. [19] proposed a wireless magnetoelastic immunosensor for detection of carcinoembryonic antigen, a potential biomarker for gastric, pancreatic, lung, and other carcinomas.

In this paper, a passive gamma detection technology comprised of a small fielded sensor and a separate analysis package is introduced. The sensor has a cylindrical form factor, with an approximate length and diameter of 15 and 2.5 mm, respectively, and it consists of an amorphous magnetoelastic wire held in a longitudinally stressed state by a radiation-sensitive material (RSM). The RSM in the device described here is Teflon (polytetrafluoroethylene, or PTFE). The RSM will sometimes be referred to as a response material. The analysis package is built around an excitation-detection coil set. The two parts of the detector can communicate wirelessly through a metallic or nonmetallic barrier at distances up to about 25 mm. When the RSM is subjected to gamma radiation, it decomposes, relaxing the stress on the wire and lowering the wire’s magnetic permeability. The changes in permeability are observed by switching the magnetic domains in the wire and measuring the reduction in the Faraday voltage as the stress is diminished. Because RSM decomposition is irreversible, the sensor provides a cumulative value of dose and the analysis can be performed at an arbitrary time after exposure. In its present form, the system can accommodate up to four linearly-arrayed sensors, affording the possibility of discriminating between radiation types or energy.

A very similar principle of detection, and much of the same instrumentation, is used in a new chemical sensor technology known as ChIMES (chemical identification by magneto-elastic sensing) [20]. In a ChIMES device, the response material expands and imposes additional stress on the wire in the presence of a chemical target. For all of the response materials considered in [20], the expansion is reversible, enabling real-time determinations of the concentration of a target, but it is possible to make a cumulative measurement with a response material that permanently deforms upon exposure.

In the following sections, the radiation detector is described in detail and its performance is evaluated. In Section 2, the main features of the magnetoelastic wire and the RSM used to build devices are summarized, the sensor fabrication process is outlined, and the initial design of the analysis system is presented. Next, several improvements to the analysis system are described. The paper continues with the performance characteristics of two sensors prepared by different methods, and concludes with a comparison to other kinds of integrating dosimeters and ideas for future work.

## 2. Materials and Methods

### 2.1. Magnetoelastic Wire

Thin, amorphous magnetoelastic wires (or “microwires”) are available from a few commercial suppliers. Reviews of microwires, with discussions of applications, have been published by Mohri et al. [21], Calkins et al. [22], and Donald [23]. The wires are fabricated from alloys containing Fe or Co, or both, and one or more glass-forming elements like B and Si. The alloys sometimes include small amounts of elements like Al, Cr, Mn, Cu, or Nb to improve magnetic, mechanical, or anticorrosive properties [24]. The wires are manufactured through a series of rapid solidification, cold drawing, and annealing processes that result in a complex distribution of residual stresses in the axial, azimuthal, and radial directions. Vázquez [25] reports average values of the stresses in the range 50–100 MPa. The stresses couple with the magnetostrictive properties of the alloys to produce a number of remarkable features: Fe–Si–B and Co–Si–B wires with positive and negative magnetostriction, respectively, display magnetic bistability, and Co-rich Co–Fe–Si–B wires with nearly zero magnetostriction are very soft and exhibit giant magnetoimpedance [25]. Jiles [26], Vázquez and Hernando [27], and Squire et al. [28] have described the magnetic domains of the wires as mainly consisting of a single, axially-oriented core domain surrounded by many small shell domains. The shell magnetization is radial and circumferential for positive- and negative-magnetostriction alloys, respectively, whereas the core magnetization is longitudinal for both types. Adjacent shells with circumferential magnetization have opposite directions of magnetization, giving rise to a “bamboo-like” structure. The shell magnetization also is circumferential for Co–Fe wires fabricated from alloys with vanishing magnetostriction. 

The experiments reported here were performed with Co–Fe–Si–B “SENCY DC2T” wire, 100 micrometers in diameter. The wire was obtained from Unitika, Ltd., of Japan. (Unitika does not publicize the full compositions of its products.) According to the manufacturer, the wire has high permeability (~10,000 at 10 kHz), very low coercivity (0.06 Oe), and nearly zero magnetostriction. Mechanically, the wire is rather brittle and tends to fracture when folded or slightly twisted. SENCY is fabricated by ejecting a stream of molten alloy through a small nozzle into a layer of cold water in a rotating drum [29,30]. The morphological and mechanical properties of wires generated by this technique, often called the in-rotating-water spinning method, are determined by the material properties of the metal, the relative velocities of the jet and drum, the angle of the nozzle against the water surface, and the temperatures of the molten jet and the water [29]. Using the in-rotating-water spinning method, Hagiwara et al. [30,31] produced continuous amorphous wires of Fe–Si–B ternary and Fe–M–Si–B (M = Ti, V, Nb, Ta, Cr, Mo, and W) quaternary alloys. SENCY wire is now sold by the Aichi Steel Corporation, also headquartered in Japan.

### 2.2. Radiation-Sensitive Material

Sensors were fabricated using laboratory-grade PTFE as the radiation-sensitive material. PTFE was chosen because it has one of the lowest thresholds for radiation damage among polymeric materials [32] and because it becomes friable at low doses [33]. The chemical and physical effects of radiation on PTFE have been reviewed by several authors [33,34,35,36,37,38,39,40,41]. It is generally accepted that PTFE suffers chain scission and increased crystallinity when subjected to ionizing radiation below the melting point (~327 °C), accompanied by reductions in the average molecular weight, although the mechanism of reaction is disputed [34]. (At and above the melting point, PTFE appears to undergo both cross-linking and chain scission, with cross-linking being dominant [38,39]. Additionally, for alpha radiation, cross-linking occurs above doses of 1 MGy [37]). Because the intermolecular chain cohesion in PTFE is relatively weak, the mechanical properties of the polymer are strongly dependent upon molecular weight and rapidly degrade with dose [40]. Oshima et al. [41] reported that the chain-scission G-value (chemical yield per 100 eV of absorbed energy) for PTFE rises sharply with temperature, increasing from 2.5 at −196 °C to about 18 at 300 °C. 

### 2.3. Sensor Fabrication

**Figure 1:**
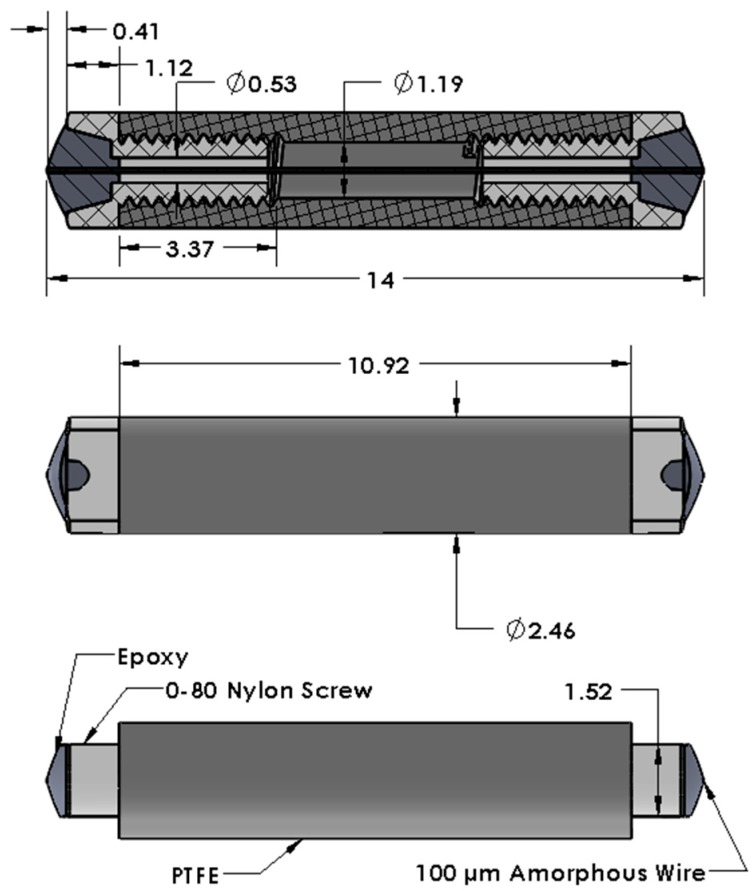
Schematics of the magnetoelastic radiation sensor (in mm).

The main challenge in sensor fabrication is securing the magnetoelastic wire to the RSM. The choice of Teflon as the response material is a complicating factor because conventional adhesives cannot be used to directly bind the wire to it. Several methods for building sensors were tested, but most were discarded because of high failure rates. The best solution involved fastening the wire to small nylon screws on each side of the RSM. In addition to providing surfaces compatible with epoxy, the screws were used to impose a variable stress upon the wire. The procedure is straightforward but requires some precision. A 1.07-mm hole is drilled through a PTFE plate on a small drill press. The drill bit is then replaced with a section of 3.02-mm-diameter stainless-steel tubing, with one end of the 0.254-mm wall sharpened to serve as a hole saw, and a concentric cut is made in the plate. After the tubing is extracted, the PTFE plug is removed, trimmed to the desired length, and tapped with a 0–80 thread on each side to a depth of 5.08 mm. Size 0-80 nylon screws with a 3.18-mm-long threaded section (McMaster-Carr, P/N 94735A701) are fully turned into the RSM. The screws have a 1.59-mm hole drilled through the centerline. One screw is then reversed 1/16th of a turn to provide a means of varying tension on the wire. A 75-mm length of 100-micrometer-diameter SENCY wire is inserted through the nylon screws, and a stop made from a blob of Hardman DOUBLE/BUBBLE Epoxy Red is deposited about 25 mm from one end of the wire. Additional blobs of epoxy are worked into the joints between the nylon screws and the wire by repeatedly moving the wire backward and forward, with care taken to limit the dispersion of the epoxy only to the sections of wire in contact with the screws. The assembly is hung vertically, with the stop on the bottom, using a set of clamps and weights that place the wire under 100 gram-force of tension while the epoxy is curing. The sensor body rests against the stop during the curing process. After the epoxy has cured, the excess wire on both sides of the device is cut and filed flush with the epoxy surface. Figure 1 displays schematics of the finished sensor.

The dimensions specified in this procedure reflect a number of practical considerations. The magnitude of the switching signal would increase if a longer wire were used, since more magnetic domains would contribute to the Faraday voltage. However, for a linear array of sensors, each sensor must be short enough to be magnetically isolated within the coil set. Similarly, the sensitivity of the device would increase with decreasing diameter of the sensor body, but the response material must be thick enough to have sufficient compressive strength to maintain stress on the wire. For long-term deployments, creep of the response material (Teflon or otherwise) or the epoxy might become a concern, and it might be necessary to develop a composite response material, or find an alternate epoxy, with more stable dimensions.

### 2.4. Analysis System

**Figure 2:**
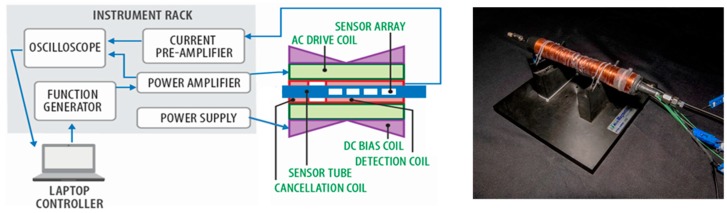
Schematic of the chemical identification by magneto-elastic sensing (ChIMES) analysis system and a photograph of the coil set.

Figure 2 presents a schematic of the analysis system built for the ChIMES technology [20] and a photograph of the excitation-detection coil set. The coil set has four components—an alternating current (AC) drive coil, a detection coil in series with a cancellation coil, and a direct current (DC) bias coil. The sensors are interrogated in a 6.35-mm-outside diameter (OD) Pyrex tube tightly mounted within the detection-cancellation coil. The schematic shows an array of four devices centered in the detection coil. In [20], the sensors were epoxied to a stiff fiber, with positions referenced to one end of the tube. In the present work, the sensors were held in a fourfold-notched cartridge fabricated from a 2.79-mm-inside diameter (ID), 3.96-mm-OD graphite tube. The cartridge permitted the sensors to be rapidly reordered or replaced between experiments and ensured that the sensor positions were reproducible. The sensors were placed flush with the left sides of the notches. There are no physical or electrical connections to the array.

The drive coil is comprised of 2556 turns of 24-gauge copper wire, is 245 mm in length, and has a magnetic induction per unit current of 13.1 mT A^−1^; the typical current in the coil is 1.4 A_pp_ at 25 Hz. During a measurement, the drive coil establishes an alternating magnetic field strong enough to switch the magnetic domains in the wire. The detection coil picks up the Faraday voltage created by fluctuations in the magnetic flux. These coils serve the same functions as the “exciting” and “response” coils in the wireless radiation sensor described by Du et al. [14]. In the configuration reported in [20], the detection and cancellation coils are wound on the same mandrel and have a total of 518 turns of 24-gauge wire. The detection coil is 144 mm in length and is separated from the 76.2-mm cancellation coil by a 7-mm gap. The cancellation coil is reverse-wired in series with the detection coil and nullifies the strong drive field within it; a rough null point is found by moving the detection-cancellation coil along the main axis of the coil set. The DC bias coil, consisting of 640 turns of 18-gauge copper wire, is used when an array of sensors is in the Pyrex tube and compensates for the tendency of all wires in the array to give overlapping signals by switching at the same time. The bias coil is wrapped so that the spacing linearly increases toward the center of the solenoid, with the winding direction reversing at the midpoint. This configuration provides an additional magnetic field with a strength that linearly varies along the sensor array. The presence of the bias field causes the switching time of each sensor to depend upon its location in the array.

The system is controlled by a LabVIEW program running on a Dell Latitude E6410 laptop computer. A function generator (Agilent U2761A) outputs a sinusoidal waveform with specified magnitude and frequency to a power amplifier (Quanser LCAM), which provides a sinusoidal current to the AC drive coil. A Sorensen XPF35-10 voltage power supply drives the bias coil. Signals from the detection/cancellation coil are processed by a low-noise current pre-amplifier (Femto DLPCA-200) and a high-speed oscilloscope (ZTEC Instruments XT4441 LXI). The oscilloscope also monitors the AC drive current through a shunt from the power amplifier. All these components are contained in an instrument rack small enough to be transported as airline carry-on luggage.

**Figure 3:**
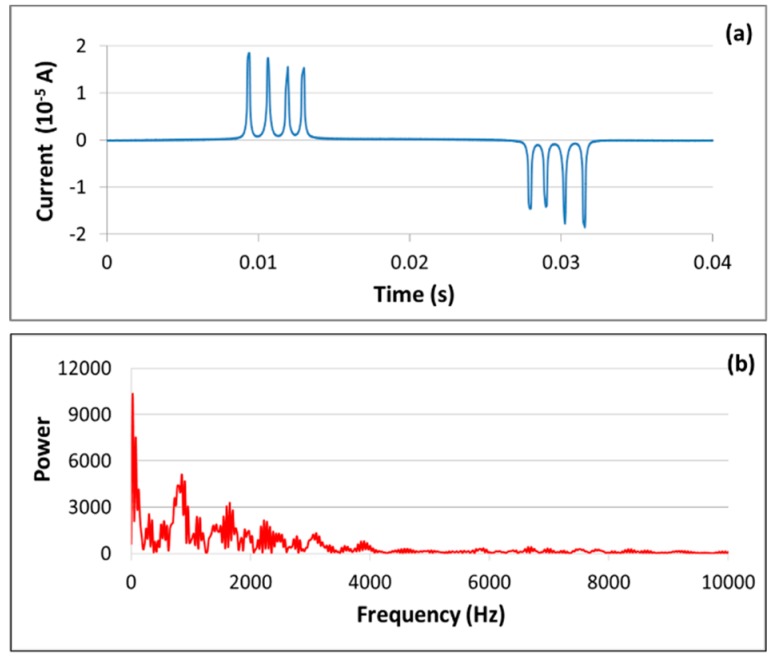
(**a**) Magnetic domain switching signals from an array of four sensors; (**b**) frequency content of the time record in part (**a**).

Figure 3a displays a current versus time plot tracing typical magnetic switching signals from an array of four sensors. The current data were measured in the detection coil and are in the range of tens of milliamperes. Current-based, low-impedance measurements were performed because it was possible to ignore the capacitance in the cable connecting the detection coil to the instrumentation package. Voltage-based, high-impedance measurements could also have been made, but in this case the cable capacitance would have degraded the frequency response. The plot has one positive and one very similar negative peak for each wire, corresponding to the oscillations of the magnetic domains as they follow the drive field. The response curve typically contains remnants of the drive field that are not removed by the cancellation coil. To ensure that these artifacts do not alter the peak heights, they are fitted to a sine function and subtracted from the baseline. During data processing, the absolute values of the magnitudes of the positive and negative switching peaks are averaged, and the response of each sensor is reported as the difference between the averages obtained before and after exposure to radiation. A total of 512 cycles are averaged for each reported current value. As will be discussed in Section 3, the four sensor positions have different gains, so the response of a sensor is averaged across all positions. Figure 3b presents the Fourier power spectrum of the time record in part (a) and will be discussed in Section 3.

The current values exhibited in Figure 3a were measured through the thin wall of the Pyrex tube containing the sensor array. It is also possible to interrogate a sensor through more substantial metallic and nonmetallic barriers, such as those that might be employed if the sensor were encapsulated. Depending upon the electromagnetic shielding properties of the barrier, measurements can be made through thicknesses extending to about 25 mm. For example, using longer wires (75 mm) and a larger coil set, readings have been taken through aluminum walls as thick as 12.7 mm. The effectiveness of an electromagnetic shielding material can be judged by the penetration depth (δ), which is defined as the depth within a material at which the magnitude of an electromagnetic field reduces to (1/e) of its value at the surface [42]. For a poor conductor, $\delta =\left(2/\sigma \right){\left(\varepsilon /\mu \right)}^{1/2}$, where σ, ε, and μ are the electrical conductivity, absolute permittivity, and absolute permeability of the material, respectively. The penetration depth of Pyrex is much larger than a meter, so the ability to communicate with a sensor in the present system mostly depends on the strengths of the excitation and response fields. For a good conductor, $\delta \approx {\left(\pi \nu \sigma \mu \right)}^{-1/2}$, where ν is the frequency of the field. The penetration depth of aluminum 6062, a possible encapsulant, is about 16 mm at 25 Hz, indicating that attenuation in the barrier is a concern. (A light metal would be used as an encapsulant since the gamma absorption cross-section of a nucleus increases approximately with the fourth power of its atomic number.) The penetration depth can be increased by operating at a lower frequency, trading off against a longer measurement time.

The sensors exhibit significant temperature sensitivities because the thermal expansivity of PTFE is much greater than that of SENCY wire. Kirby [43] reports a coefficient of linear thermal expansion of 1.24 × 10^−4^ K^−1^ for PTFE near room temperature, and internal (unpublished) data suggest a value of the order of 10^−5^ K^−1^ for SENCY. Measurements done with the magnetoelastic chemical sensor indicated a temperature coefficient of about 0.3 μA K^−1^ for a polymer-based response material [20]. Using a type-T thermocouple within the Pyrex tube, the operating temperature during the present experiments was determined to be about 44 °C.

### 2.5. Sensors and Exposures

**Figure 4:**
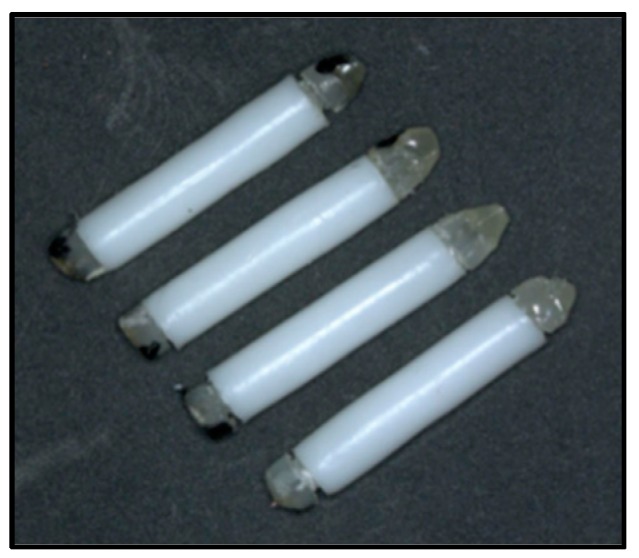
First sensor set (S1).

Two sets of sensors were built for the performance evaluations. The first set (S1, Figure 4) consisted of four sensors prepared according to one of the original assembly methods, with lengths in the range of 15.0 ± 0.2 mm and diameters in the range of 2.45 ± 0.05 mm. Two of these devices, along with a replacement, failed when additional loading was attempted by twisting one screw 1/16th of a turn counter-clockwise. The remaining two sensors, designated S1.1 and S1.4, were loaded successfully. S1.1 was subjected to radiation exposures, and S1.4 was used as a control. The second set of sensors (S2) was assembled following the procedure in Section 2.3 and consisted of six devices with lengths of 14.0 ± 0.3 mm and diameters of 2.45 ± 0.05 mm. All sensors in this group, designated S2.1 to S2.6, were intact. Sensor S2.5 was used in the performance tests, with S2.1 as control. S2.6 was employed as a control for S1.1 after the adhesive joint in S1.4 failed; at that point, the dose in S1.1 from two exposures amounted to only about 1 Gy. The control sensors were not irradiated and were mainly used to account for the temperature sensitivity described at the end of the last section.

Because a pure gamma source of sufficient power was not available, a Phillips MGC-30 X-ray generator operating in fluoroscopy mode was used to irradiate the sensors. The generator was set to a 400-kV beam at 10.5 mA, and the dose rate was calibrated with an electrometer and ionization chamber over a range of irradiation times. The peak and average energies were about 450 and 220 keV, respectively. The sensors were placed 11.43 cm from the aperture at the beam centerline, for an exposure rate of 13.4 R/s. The exposure times were much longer than the voltage ramp-up times, permitting an assumption of a single distribution of beam energies. The exposures were performed in air in an X-ray vault with controlled humidity. 

Sensors S1.1 and S2.5 were given incremental doses reaching 7 kGy for S1.1 and 6 kGy for S2.5. These doses are far above those associated with background radiation. As noted above, the responses of the devices and their controls were averaged across the four sensor positions designated below as P1 to P4. After each rotation, at least 10 min was allotted for the responses to stabilize. The results are reported as the response of sensor S1.1 divided by the response of control S2.6 and the response of sensor S2.5 divided by the response of control S2.1 (called S1.1/S2.6 and S2.5/S2.1 for convenience). An effort was made to maintain similar thermal histories for the test sensors and their controls. For a given dose level, the responses of S1.1 were always larger than those of S2.5 due to S1.1 being longer than S2.5. On average, the 8% longer length of S1.1 led to a 32% increase in response. 

## 3. Improvements to the Analysis System

As mentioned in the introduction, the analysis system for the magnetoelastic radiation sensor is very similar to that used in [20]. This system was refined several times during development of the ChIMES technology but is still considered immature. Much of the work reported here was concerned with improving the sensitivity of the detection coil and the consistency of the gain across the sensor positions.

The combined detection-cancellation coil in [20] contains 518 turns of 24-gauge wire, with one layer of windings for the detection coil and two layers for the cancellation coil. The detection coil sits at the approximate center of the drive coil, with the cancellation coil alongside. Ideally, the series impedance of the two coils would match the 50-Ω input impedance of the current preamplifier. In practice, the series impedance is only 0.98 Ω, suggesting that the sensitivity of the detection coil could be increased by adding more turns of wire. However, as the number of turns increases, the frequency response decreases. Figure 3b indicates that the frequency content of the magnetic domain switching curve is bounded at about 10 kHz, so the bandwidth of the new detection-cancellation coil should be slightly greater than this value. Because there is limited space between the drive and detection-cancellation coils, the additional turns require a reduction in wire gauge and changes in geometry.

**Figure 5:**
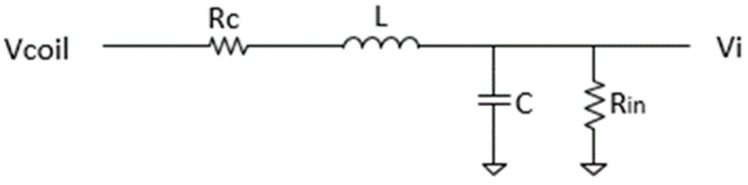
RLC model of the detection-cancellation coil.

If the detection-cancellation coil is modeled as shown in Figure 5, the transfer function of preamplifier input current (*i_in_*) to voltage induced in the detection coil (*V_coil_*) can be written as

$$\frac{i_{in}}{V_{coil}}\;=\;\frac{\frac{1\;+\;\frac{R_{c}}{R_{in}}\;-\;w^{2}LC}{R_{in}}\;+\;C\left(R_{c}C\;+\;\frac{L}{R_{in}}\right)w^{2}}{{\left(1\;+\;\frac{R_{c}}{R_{in}}\;-\;w^{2}LC\right)}^{2}\;+\;{\left(R_{c}C\;+\;\frac{L}{R_{in}}\right)}^{2}w^{2}}+j\frac{Cw\left(1\;+\;\frac{R_{c}}{R_{in}}\;-\;w^{2}LC\right)\;-\;\frac{(R_{c}C\;+\;\frac{L}{R_{in}})w}{R_{in}}}{{\left(1\;+\;\frac{R_{c}}{R_{in}}\;-\;w^{2}LC\right)}^{2}\;+\;{\left(R_{c}C\;+\;\frac{L}{R_{in}}\right)}^{2}w^{2}}$$

where *R_c_* and *R_in_* are the coil and preamplifier input resistances, respectively; *L* is the coil inductance; and *C* is the preamplifier input capacitance. This equation was solved graphically to furnish 3 db roll-off frequencies for a series of new designs for the detection-cancellation coil. Some characteristics of these designs and the results of the frequency determinations are given in Table 1. The top line in the table represents the ChIMES configuration. All new designs have two layers for the detection coil and three layers for the cancellation coil, and all are “split,” meaning that the cancellation coil is divided into a 56.4-mm section on one side of the detection coil and a 57.7-mm section on the other. The split configuration is more space-efficient for the cancellation coil and it permits a second layer of windings on the detection coil. In addition, it enables one to more precisely nullify the drive field within the detection coil. For the cancellation coil to completely eliminate the drive field within the detection coil, the two coils should have approximately the same number of turns within the drive field. If the turn density on one side of the cancellation coil is slightly different from that on the other side, the total number of turns in the drive field can be modified by small adjustments in the position of the cancellation coil with respect to the center of the drive coil.

**Figure 6:**
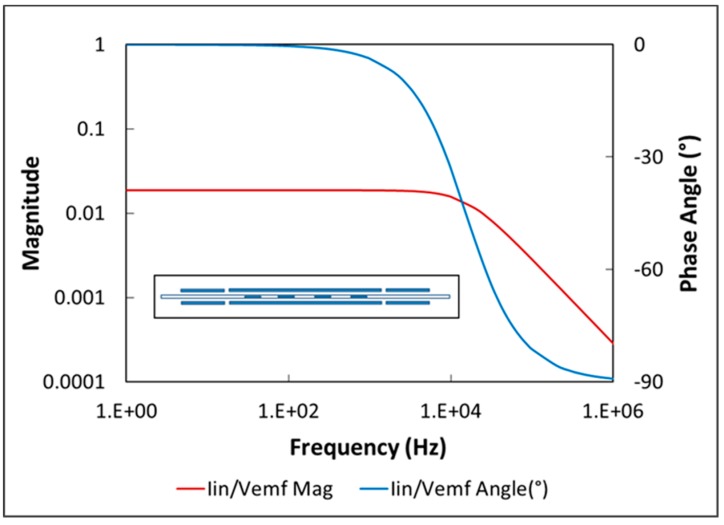
Bode plot and geometry (inset) of the new detection-cancellation coil.

The roll-off frequencies in Table 1 indicate that the configurations with a wire gauge of 30 or less would capture the frequency content of the switching signal. (The roll-off frequency of the original configuration is about 133 kHz, much larger than necessary.) The option with 28-gauge wire, and a roll-off frequency of about 16 kHz, was chosen as a trade-off between frequency response and mechanical strength of the wire. A Bode plot of the transfer function for this configuration (Figure 6) demonstrates that the magnitude and phase angle are both very flat through at least 1 kHz. The inset in this figure displays the symmetric detection-cancellation coil geometry. The new design resulted in a 3.3-fold increase in sensitivity over the detection-cancellation coil used in the ChIMES work.

A second advantage of using the new detection-cancellation coil is that the sensor array is centered in the coil set rather than skewed to one side. The centered location leads to more consistent gains across the sensor positions. To demonstrate this, the gains produced by the old and new geometries were determined by rotating a set of zero-dose sensors through the four positions on the notched cartridge. From one side of the coil set to the other, the gains were 5.7%, 2.2%, 0.45%, and −8.3% removed from the average for the old configuration; and −3.7%, 2.8%, 2.4%, and −1.6% removed from the average for the new configuration. The remaining inconsistency was probably mostly due to the curvature of the composite magnetic field, especially at the ends of the coil set.

**Figure 7:**
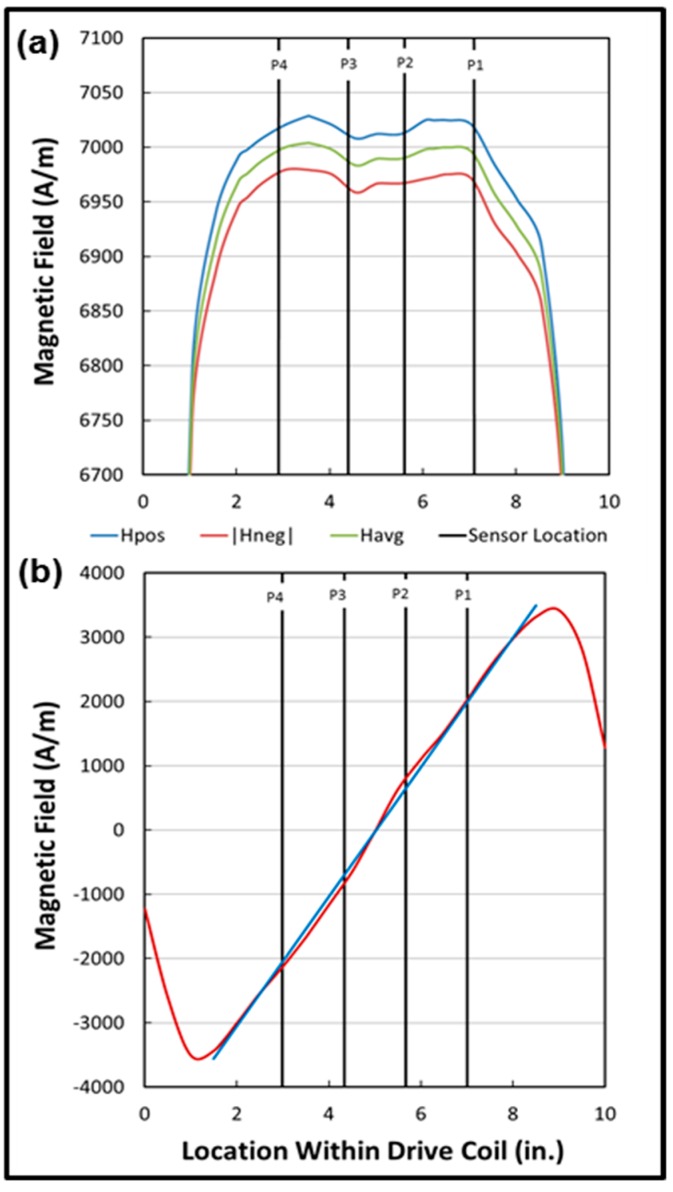
Sensor positions in the (**a**) drive and (**b**) bias fields with the new detection-cancellation coil.

To visualize the magnetic environment of the sensors, the magnetic field associated with the AC drive coil was measured by passing a 3.175-mm-ID, 3.53-mm-OD coil with 70 turns of 32-gauge wire through the Pyrex tube [45]. The size of this probe was roughly the same as that of a sensor. The coil was wrapped on a 3.175-mm polyether ether ketone (PEEK) tube, with the twisted leads brought out through the center of the tube. Figure 7a displays the positive field in blue, the absolute value of the negative field in red, and the average of the two across the coil set in green. (The positive and negative fields are themselves averages of 512 scans.) The sensor positions are indicated in black. The fields are roughly symmetrical with respect to the center of the coil set and relatively flat in the region occupied by the array. The sensor position with the gain most removed from the average (P1) is very close to the rapidly falling regions of the positive and negative fields. 

Similarly, the magnetic field associated with the bias coil was determined by passing a DC magnetometer through the coil set. The magnetometer was a Hall probe supplied by AlphaLab, Inc., and it was mounted on the end of a 6.35-mm glass tube. The results are shown in Figure 7b, with the DC magnetic field in red, a first-order least-squares fit in blue, and the sensor positions in black. The zero point of the bias field is located at the center of the array. (In the original design, because of the asymmetry of the detection-cancellation coil, the zero point fell on sensor position P3.) The deviations from the fitted line are approximately symmetrical about the zero point and amount to less than 5% of the largest bias field. These small variations should slightly affect the spacing, but not the magnitudes, of the switching peaks since the bias only serves to shift the peaks along the time dimension. The peaks typically are well separated, as illustrated in Figure 3a.

## 4. Sensor Performance

**Figure 8:**
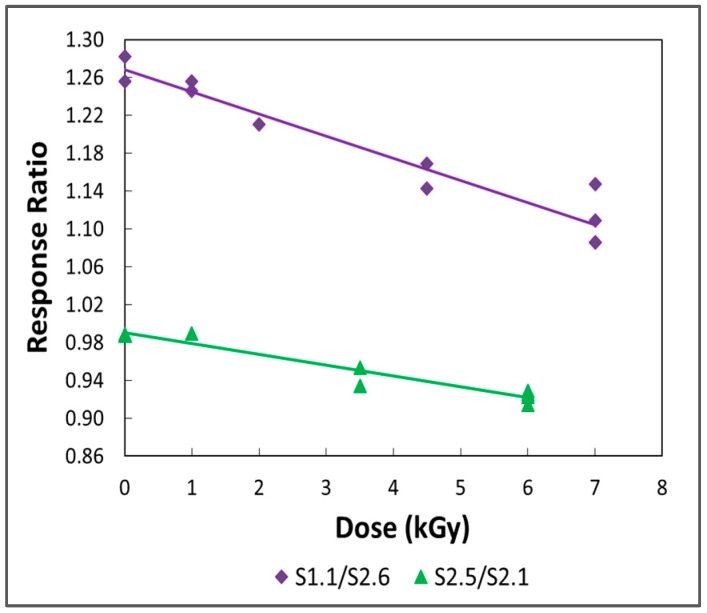
Radiation performances of two magneto-elastic sensors. (The vertical scale is test sensor response divided by control sensor response.).

Figure 8 displays plots of response ratios S1.1/S2.6 and S2.5/S2.1 against radiation dose. The plots are quite linear—the correlation coefficients (r^2^) of first-order fitted curves are 0.932 and 0.931, respectively—and they do not exhibit response thresholds at low doses. The offset in the curves is a result of the differing lengths of the sensors. The negative slopes indicate that the longitudinal stresses on the wires in sensors S1.1 and S2.5 are being steadily relaxed with increasing dose and that these changes are persistent. (Recall that S2.6 and S2.1 are control units that were not irradiated.) The linearity of the plots suggests that doses of 7 kGy or less fall on the initial part of an extensive response curve. This is consistent with the results of Lappan et al. [46], who used ^19^F solid-state NMR and IR spectroscopy to investigate modifications in the chemical structure of PTFE induced by electron-beam irradiation in air at room temperature. Lappan et al. [46] found that the number-average molecular weight of the polymer decreased with dose to at least 4 MGy, and that the molecular weight dropped rapidly at low doses. In addition, they confirmed that chain scission was the predominant degradation process under their experimental conditions. As discussed in Section 2.2, the mechanical properties of PTFE are strongly dependent upon molecular weight and rapidly deteriorate with dose [40]. At doses higher than those employed here, it is anticipated that the response curves will display increasing nonlinearity. It should also be mentioned that the decreasing stresses may reflect radiation-induced compromise of the epoxy or of the adhesive joints between the wires and nylon screws.

The absence of response thresholds in Figure 8 reflects the granularity of the measurements, since there must be a minimum dose associated with a perceptible change in the magnetic state of the wire. The smallest dose plotted in the figure is about 1 kGy. This is a somewhat coarse increment, and it is likely that the minimum observable response corresponds to a dose well below it. The sensitivity and linear range of the magnetoelastic sensor are compared below with the characteristics of other types of dosimeters.

The S1.1/S2.6 fitted line is more than twice as steep as the S2.5/S2.1 line: the slopes are −0.0234 and −0.0114/kGy, respectively. The bodies of the two test sensors were made from the same block of PTFE, so it is unlikely that inconsistencies in source material account for the disparity in sensitivity. However, differences in the fabrication procedures used for S1.1 and S2.5 may be the cause. S1.1 was prepared by one of the discarded methods, in which the wire was epoxied to the two sides of the RSM in two separate operations. For the first side, the epoxy was worked into the joint by moving the wire backward and forward through the screw, and then the assembly was permitted to cure with no load on the wire. For the second side, the epoxy was dispersed by repeatedly loosening and tightening the screw. It is likely that less epoxy was deposited in the critical region in the second case, leading to a relatively weak attachment that may have distorted or slipped to a greater extent under irradiation. S2.5, on the other hand, was built by the optimized procedure described in Section 2.3, in which both adhesive joints were prepared at the same time through the moving-wire technique.

At a given dose, the response of a sensor usually fades, sometimes reversibly, during interrogation, suggesting that one or more of the materials in the device is undergoing creep at the operating temperature of 44 °C. It is likely that the epoxy is deforming—the Vicat B50 softening points of PTFE [47] and Nylon [48] are over 100 °C, whereas the lap shear strength of Hardman DOUBLE/BUBBLE epoxy decreases from 20.7 to 16.5 MPa as the temperature rises from 25 to 38 °C [49]. The rate of fall-off slows after some time in the coil set, so some conditioning at an elevated temperature may be necessary for a fielded sensor. In addition, the response decreases irreversibly during storage at room temperature. Figure 8 shows groups of measurements at most doses. The individual points in these groups were typically taken at one-week intervals; the values fall as the storage time increases. The fading was as much as 4% over one week. This behavior may indicate that there is both a prompt and a delayed response to radiation, and that the delayed response occurs over a period of weeks. Because the main chain of PTFE is largely covered by fluorine atoms, and free radicals produced on the chain by irradiation experience great steric hindrance [40], it is unlikely that the fragments generated by scission will recombine and lead to the self-repairing behavior exhibited by, for example, l-α-alanine [50]. Additionally, the magnetoelastic sensor does not display the fading due to charge recombination and trapping that is characteristic of semiconductor diode detectors [51].

## 5. Discussion and Conclusions

A passive gamma detection technology, consisting of a small fielded sensor, with no powered or moving parts, and a separate electronics and analysis package, has been demonstrated. The principle of detection relies on changes in the permeability of a magnetoelastic wire, mediated by a response material that alters longitudinal stress imposed upon the wire in the presence of radiation. Prototypical devices were constructed with Teflon (polytetrafluoroethylene, or PTFE), which mechanically degrades upon irradiation, as the response material. The magnetic basis of measurement enables the two components of the detector to communicate wirelessly through a metallic or nonmetallic barrier at distances up to about 25 mm. Because the reactions of the response material are irreversible, the device provides a cumulative measure of dose and the analysis can be performed at an arbitrary time after exposure. With clever choices of response materials, an array of sensors provides the possibility of discriminating between radiation types or energy.

In initial experiments against radiation with peak and average energies of 450 and 220 keV, respectively, the sensors exhibited linear responses across the full range of imposed doses (0–7 kGy), with fading of a few percent per week. The sensitivity was somewhere below 1 kGy. When assessing the performances of these devices, it should be kept in mind that they operate in a very different manner from conventional passive radiation detectors. Still, it can be insightful to compare them to other integrating dosimeters like alanine pellets and TLDs. A number of companies offer alanine pellet dosimeters. The FWT-50 Series dosimeter [52], manufactured by Far West Technology, Inc. (Goleta, CA, USA), has a linear response for doses up to 3 kGy and fading of about 1% a year. Bhatta and Kulkarni [5] tabulate the sensitivity, linear range, and fading of TLDs based on fourteen thermoluminescent materials, with sensitivity given relative to the TLD-100 dosimeter (LiF:Mg, Ti). For the TLD-100, Bhatta and Kulkarni [5] quote detection thresholds of several tens of microGrays from several manufacturers; in addition, Moor et al. [53] found a detection threshold of 70 μGy against ^90^Sr/^90^Y radiation, and Bauk et al. [54] reported a threshold of 300 μGy against 28 keV X-rays. The fourteen materials have a minimum linear range of 0.1 mGy–0.5 Gy (BeO) and a maximum linear range of 10 μGy–1 kGy (Li_2_B_4_O_7_:Cu), and minimum and maximum relative sensitivities of 0.40 (Li_2_B_4_O_7_:Mn) and 60 (Al_2_O_3_:C), respectively. The fadings range from negligible (LiF:Mg, Cu, Si) to 16% over two weeks (CaF_2_:Mn).

As work on the sensor continues, one of the main interests will be a better determination of its performance characteristics, especially at low doses. Other efforts will concern the synthesis of response materials with lower thresholds for radiation damage, and perhaps the development of magnetoelastic wires that are less brittle. Automation of the fabrication process by, for instance, stamping the sensor bodies from a slab of response material or building them with an additive manufacturing process should improve the uniformity of the devices. In addition, the use of a response material more compatible with adhesives than PTFE would eliminate the need for the adjustment screws. With these changes, it is possible that sensors could be made that do not require pairing with controls.

Finally, whereas all results have been presented for an RSM that degrades upon exposure to radiation, and for a sensor that functions as a dosimeter, it should be noted that other configurations are possible. Another device that records cumulative dose could be built if the wire is slightly prestressed and mounted in an RSM that irreversibly expands upon irradiation. Alternatively, a resettable version could be constructed if an RSM could be identified that expands upon irradiation and relaxes during a post-exposure treatment like annealing.

## 6. Patent

Lamberti, V.E.; Howell, L.N., Jr.; Mee, D.K.; Kress, R.L. Wireless radiation sensor. U.S. Patent 9,411,069, 9 August 2016.

## Figures and Tables

**Table 1:** Characteristics of new designs for the detection (D)-cancellation (C) coil.

Wire Gauge	Wire Resistance [44] (Ω/1000 ft)	Geometry	D + C Turns	L (μH)	R (Ω)	Coil Layers (D, C)	C Coil Diameter (mm)	C Coil Length (mm)	3 db Roll-Over Frequency (Hz)
**24**	25.67	Not Split	518	61	1.00	1, 2	8.69	73.7	133,478
**24**	25.67	Split	1120	275	2.36	2, 3	9.88	114	30,472
**26**	40.81	Split	1380	390	2.81	2, 3	9.25	114	21,813
**28**	64.90	Split	1693	554	3.35	2, 3	8.74	114	15,713
**30**	103.2	Split	2058	784	3.98	2, 3	8.33	114	11,245
**32**	164.1	Split	2488	1103	4.72	2, 3	8.03	114	7905
